# Crystal structure of 4-nitro-*N*-[(pyridin-2-yl)methyl­idene]aniline

**DOI:** 10.1107/S2056989015016928

**Published:** 2015-09-17

**Authors:** Watcharin Saphu, Kittipong Chainok

**Affiliations:** a Department of Chemistry, Faculty of Science, Naresuan University, Muang, Phitsanulok 65000, Thailand; b Department of Physics, Faculty of Science and Technology, Thammasat University, Khlong Luang, Pathum Thani 12120, Thailand

**Keywords:** crystal structure, hydrogen bonds, Schiff base, π–π stacking

## Abstract

The title compound, C_12_H_9_N_3_O_2_, adopts an *E* conformation at the imine double bond. The pyridyl ring makes a dihedral angle of 47.78 (5)° with the benzene ring, indicating the mol­ecule is twisted. In the crystal, mol­ecules are π–π stacked into columns parallel to [100], with an inter­planar separation of 3.8537 (8) Å, corresponding to the length of the *a* axis. The chains are further linked *via* weak C—H⋯O and C—H⋯N hydrogen bonds, forming two-dimensional sheets parallel to (010). The sheets interact by van der Waals inter­actions.

## Related literature   

For related crystal structures, see: Zheng & Lee (2012 ▸); Marjani *et al.* (2011 ▸); Tzimopoulos *et al.* (2010 ▸); Heinze & Bueno Toro (2004 ▸).
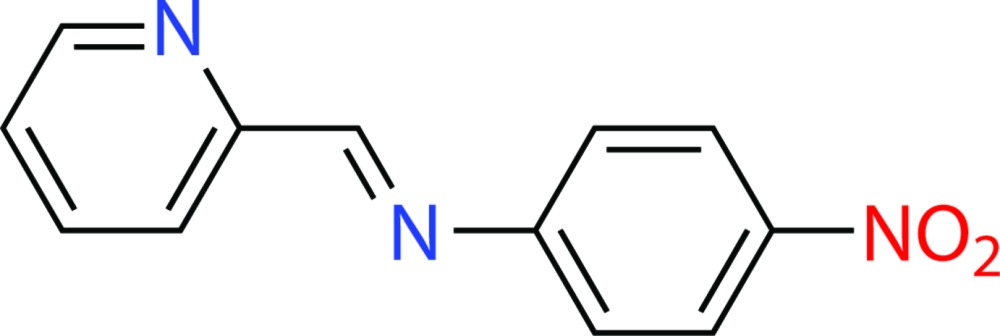


## Experimental   

### Crystal data  

C_12_H_9_N_3_O_2_
*M* *_r_* = 227.22
Monoclinic, 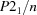

*a* = 3.8573 (8) Å
*b* = 20.334 (4) Å
*c* = 13.629 (3) Å
β = 90.57 (3)°
*V* = 1068.9 (4) Å^3^
*Z* = 4
Mo *K*α radiation
μ = 0.10 mm^−1^
*T* = 296 K
0.22 × 0.14 × 0.14 mm

### Data collection  

Bruker D8 QUEST CMOS diffractometer
Absorption correction: multi-scan (*SADABS*; Bruker, 2014 ▸) *T*_min_ = 0.983, *T*_max_ = 0.986
4963 measured reflections
2729 independent reflections
1389 reflections with *I* > 2σ(*I*)
*R*_int_ = 0.044

### Refinement  

*R*[*F* ^2^ > 2σ(*F* ^2^)] = 0.055
*wR*(*F* ^2^) = 0.146
*S* = 0.96
2729 reflections
154 parameters
H-atom parameters constrained
Δρ_max_ = 0.15 e Å^−3^
Δρ_min_ = −0.18 e Å^−3^

### 

Data collection: *APEX2* (Bruker, 2014 ▸); cell refinement: *SAINT* (Bruker, 2014 ▸); data reduction: *SAINT*; program(s) used to solve structure: *SHELXT* (Sheldrick, 2015*a* ▸); program(s) used to refine structure: *SHELXL2015* (Sheldrick, 2015*b* ▸); molecular graphics: *OLEX2* (Dolomanov *et al.*, 2009 ▸); software used to prepare material for publication: *publCIF* (Westrip, 2010 ▸) and *enCIFer* (Allen *et al.*, 2004 ▸).

## Supplementary Material

Crystal structure: contains datablock(s) I. DOI: 10.1107/S2056989015016928/tk5383sup1.cif

Structure factors: contains datablock(s) I. DOI: 10.1107/S2056989015016928/tk5383Isup2.hkl

Supporting information file. DOI: 10.1107/S2056989015016928/tk5383Isup3.cdx

Supporting information file. DOI: 10.1107/S2056989015016928/tk5383Isup4.cml

**Fig. 1.**
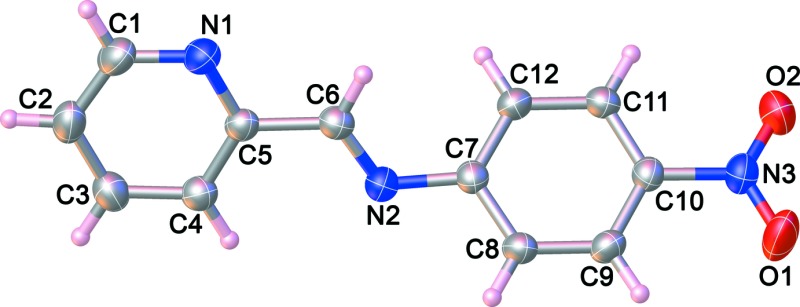
The molecular structure of (I), showing 35% probability displacement ellipsoids nd atom labels.

. DOI: 10.1107/S2056989015016928/tk5383fig1.tif
The mol­ecular structure of (I), showing 35% probability displacement ellipsoids nd atom labels.

**Fig. 2.**
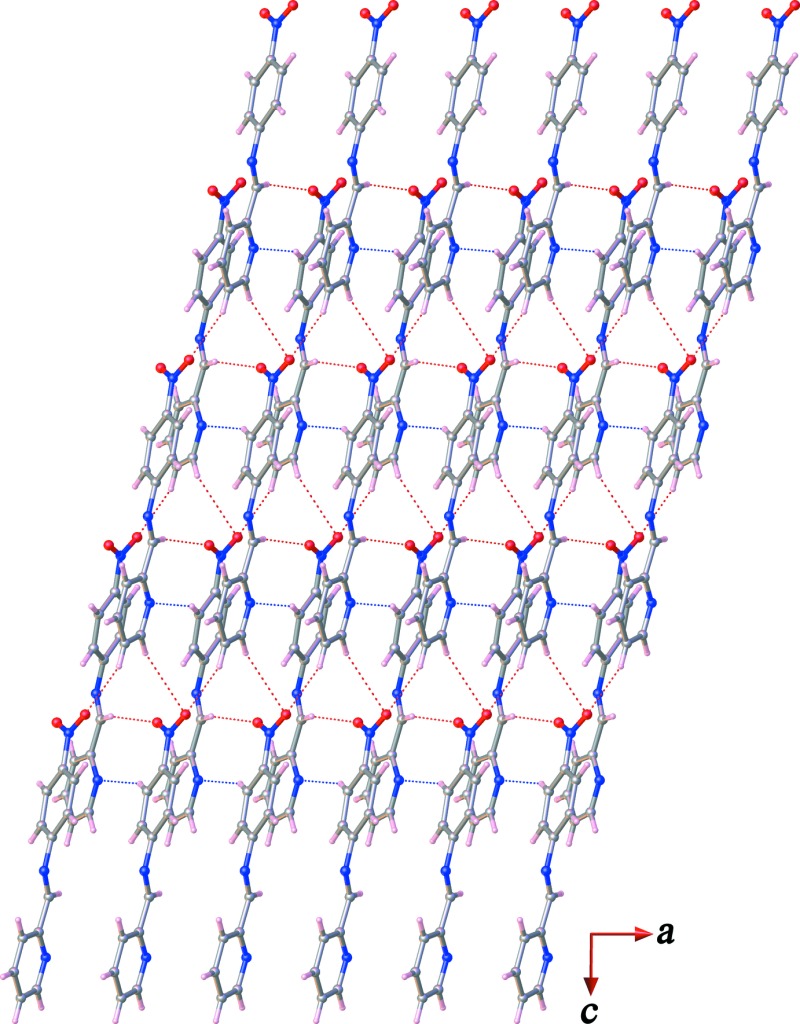
A packing view of (I) along (010). Hydrogen bonds are shown as dashed lines.

. DOI: 10.1107/S2056989015016928/tk5383fig2.tif
A packing view of (I) along (010). Hydrogen bonds are shown as dashed lines.

CCDC reference: 1423407

Additional supporting information:  crystallographic information; 3D view; checkCIF report

## Figures and Tables

**Table 1:** Hydrogen-bond geometry (, )

*D*H*A*	*D*H	H*A*	*D* *A*	*D*H*A*
C2H2O1^i^	0.93	2.65	3.343(3)	132
C6H6O2^ii^	0.93	2.65	3.527(2)	158
C11H11N1^iii^	0.93	2.60	3.465(2)	155

Symmetry codes: (i) 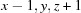; (ii) 
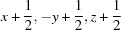
; (iii) 
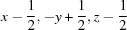
.

## supplementary crystallographic information

### S1. Synthesis and crystallization

At room temperature, 2-pyridine­carboxaldehyde (1.90 ml, 0.02 mol) was added to a benzene solution (100 ml) of 4-nitro­aniline (2.76 g, 0.02 mol), with a few drops of acetic acid added as catalyst. The reaction mixture was stirred under reflux at 110 °C. After 6 h of reflux, the yellow solution was neutralized with Na_2_CO_3_ (2 mmol), filtered, and concentrated to dryness *in vacuo*. The residue was recrystallized from a mixture of CH_2_Cl_2_ and petroleum ether (2:1, *v*/*v*) to give light-yellow crystalline solid of (I).

### S2. Refinement

The C–bound hydrogen atoms were placed in geometrically idealized positions and constrained to ride on their parent atom positions with a C—H distances of 0.93 Å, and with *U*_iso_(H) = 1.2*U*_eq_(C).

### Crystal data

**Table:**

C_12_H_9_N_3_O_2_	*F*(000) = 472
*M**_r_* = 227.22	*D*_x_ = 1.412 Mg m^−^^3^
Monoclinic, *P*2_1_/*n*	Mo *K*α radiation, λ = 0.71073 Å
*a* = 3.8573 (8) Å	θ = 3.4–25.0°
*b* = 20.334 (4) Å	µ = 0.10 mm^−^^1^
*c* = 13.629 (3) Å	*T* = 296 K
β = 90.57 (3)°	Block, light-yellow
*V* = 1068.9 (4) Å^3^	0.22 × 0.14 × 0.14 mm
*Z* = 4	

### Data collection

**Table:**

Bruker D8 QUEST CMOS diffractometer	1389 reflections with *I* > 2σ(*I*)
Graphite monochromator	*R*_int_ = 0.044
ω scans	θ_max_ = 28.8°, θ_min_ = 3.4°
Absorption correction: multi-scan (*SADABS*; Bruker, 2014)	*h* = −5→5
*T*_min_ = 0.983, *T*_max_ = 0.986	*k* = −27→26
4963 measured reflections	*l* = −18→18
2729 independent reflections	

### Refinement

**Table:**

Refinement on *F*^2^	Primary atom site location: structure-invariant direct methods
Least-squares matrix: full	Hydrogen site location: inferred from neighbouring sites
*R*[*F*^2^ > 2σ(*F*^2^)] = 0.055	H-atom parameters constrained
*wR*(*F*^2^) = 0.146	*w* = 1/[σ^2^(*F*_o_^2^) + (0.0721*P*)^2^] where *P* = (*F*_o_^2^ + 2*F*_c_^2^)/3
*S* = 0.96	(Δ/σ)_max_ < 0.001
2729 reflections	Δρ_max_ = 0.15 e Å^−^^3^
154 parameters	Δρ_min_ = −0.18 e Å^−^^3^
0 restraints	

### Special details

**Table:**

Geometry. All e.s.d.'s (except the e.s.d. in the dihedral angle between two l.s. planes) are estimated using the full covariance matrix. The cell e.s.d.'s are taken into account individually in the estimation of e.s.d.'s in distances, angles and torsion angles; correlations between e.s.d.'s in cell parameters are only used when they are defined by crystal symmetry. An approximate (isotropic) treatment of cell e.s.d.'s is used for estimating e.s.d.'s involving l.s. planes.

### Fractional atomic coordinates and isotropic or equivalent isotropic displacement parameters (Å^2^)

**Table:**

	*x*	*y*	*z*	*U*_iso_*/*U*_eq_	
O1	1.1083 (5)	0.13951 (9)	0.12132 (11)	0.0956 (6)	
O2	0.8270 (5)	0.22866 (8)	0.14401 (10)	0.0803 (5)	
N1	0.7398 (4)	0.15745 (8)	0.80644 (11)	0.0590 (5)	
N2	0.7157 (4)	0.09779 (7)	0.56225 (11)	0.0514 (4)	
N3	0.9470 (5)	0.17696 (9)	0.17367 (11)	0.0593 (5)	
C1	0.6529 (6)	0.14026 (10)	0.89705 (15)	0.0665 (6)	
H1	0.7013	0.1697	0.9476	0.080*	
C2	0.4961 (6)	0.08178 (10)	0.92062 (15)	0.0649 (6)	
H2	0.4409	0.0721	0.9853	0.078*	
C3	0.4229 (5)	0.03823 (10)	0.84726 (14)	0.0608 (5)	
H3	0.3171	−0.0018	0.8611	0.073*	
C4	0.5083 (5)	0.05437 (9)	0.75229 (13)	0.0515 (5)	
H4	0.4605	0.0256	0.7008	0.062*	
C5	0.6660 (5)	0.11411 (8)	0.73519 (12)	0.0460 (4)	
C6	0.7641 (5)	0.13469 (8)	0.63552 (13)	0.0488 (5)	
H6	0.8641	0.1758	0.6265	0.059*	
C7	0.7881 (5)	0.12104 (8)	0.46689 (12)	0.0453 (4)	
C8	0.9466 (5)	0.07832 (8)	0.40174 (13)	0.0508 (5)	
H8	1.0149	0.0367	0.4228	0.061*	
C9	1.0034 (5)	0.09701 (9)	0.30649 (13)	0.0506 (5)	
H9	1.1141	0.0688	0.2632	0.061*	
C10	0.8940 (5)	0.15815 (8)	0.27591 (12)	0.0459 (5)	
C11	0.7340 (5)	0.20177 (8)	0.33848 (13)	0.0489 (5)	
H11	0.6609	0.2428	0.3163	0.059*	
C12	0.6854 (5)	0.18309 (8)	0.43442 (13)	0.0494 (5)	
H12	0.5828	0.2122	0.4780	0.059*	

### Atomic displacement parameters (Å^2^)

**Table:**

	*U*^11^	*U*^22^	*U*^33^	*U*^12^	*U*^13^	*U*^23^
O1	0.1297 (16)	0.1088 (13)	0.0488 (9)	0.0354 (12)	0.0238 (9)	−0.0004 (9)
O2	0.1152 (14)	0.0682 (10)	0.0575 (9)	0.0082 (9)	0.0074 (8)	0.0176 (8)
N1	0.0777 (12)	0.0518 (9)	0.0473 (9)	0.0002 (8)	−0.0048 (8)	−0.0048 (7)
N2	0.0641 (11)	0.0471 (8)	0.0431 (9)	−0.0009 (7)	0.0031 (7)	−0.0005 (7)
N3	0.0723 (12)	0.0635 (11)	0.0421 (9)	−0.0019 (9)	0.0024 (8)	0.0009 (8)
C1	0.0924 (17)	0.0614 (13)	0.0455 (12)	0.0092 (12)	−0.0062 (11)	−0.0079 (9)
C2	0.0821 (16)	0.0690 (14)	0.0438 (11)	0.0121 (12)	0.0072 (10)	0.0064 (10)
C3	0.0689 (14)	0.0559 (11)	0.0576 (13)	−0.0003 (10)	0.0044 (10)	0.0096 (10)
C4	0.0591 (12)	0.0471 (11)	0.0483 (11)	0.0026 (9)	−0.0018 (8)	−0.0016 (8)
C5	0.0531 (11)	0.0421 (9)	0.0427 (10)	0.0063 (8)	−0.0015 (8)	−0.0017 (8)
C6	0.0544 (12)	0.0422 (9)	0.0497 (11)	−0.0014 (8)	−0.0001 (9)	0.0002 (8)
C7	0.0542 (11)	0.0413 (9)	0.0402 (10)	−0.0055 (8)	−0.0001 (8)	−0.0005 (7)
C8	0.0680 (13)	0.0385 (9)	0.0459 (10)	0.0044 (9)	−0.0015 (9)	−0.0013 (8)
C9	0.0595 (12)	0.0473 (10)	0.0449 (10)	0.0058 (9)	0.0026 (9)	−0.0085 (8)
C10	0.0529 (12)	0.0460 (10)	0.0390 (10)	−0.0046 (8)	0.0003 (8)	−0.0006 (8)
C11	0.0615 (12)	0.0375 (9)	0.0476 (11)	0.0013 (8)	0.0000 (8)	0.0000 (8)
C12	0.0608 (12)	0.0423 (10)	0.0452 (10)	0.0018 (9)	0.0053 (8)	−0.0055 (8)

### Geometric parameters (Å, º)

**Table:**

O1—N3	1.219 (2)	C4—C5	1.380 (3)
O2—N3	1.216 (2)	C5—C6	1.474 (3)
N1—C1	1.330 (3)	C6—H6	0.9300
N1—C5	1.340 (2)	C7—C8	1.388 (2)
N2—C6	1.262 (2)	C7—C12	1.393 (2)
N2—C7	1.413 (2)	C8—H8	0.9300
N3—C10	1.461 (2)	C8—C9	1.372 (3)
C1—H1	0.9300	C9—H9	0.9300
C1—C2	1.374 (3)	C9—C10	1.376 (2)
C2—H2	0.9300	C10—C11	1.380 (2)
C2—C3	1.363 (3)	C11—H11	0.9300
C3—H3	0.9300	C11—C12	1.376 (3)
C3—C4	1.378 (3)	C12—H12	0.9300
C4—H4	0.9300		
			
C1—N1—C5	116.52 (17)	N2—C6—H6	119.2
C6—N2—C7	119.98 (15)	C5—C6—H6	119.2
O1—N3—C10	118.14 (17)	C8—C7—N2	118.10 (15)
O2—N3—O1	122.71 (16)	C8—C7—C12	119.28 (16)
O2—N3—C10	119.14 (17)	C12—C7—N2	122.47 (16)
N1—C1—H1	118.0	C7—C8—H8	119.8
N1—C1—C2	124.10 (19)	C9—C8—C7	120.47 (16)
C2—C1—H1	118.0	C9—C8—H8	119.8
C1—C2—H2	120.7	C8—C9—H9	120.5
C3—C2—C1	118.63 (19)	C8—C9—C10	119.04 (16)
C3—C2—H2	120.7	C10—C9—H9	120.5
C2—C3—H3	120.5	C9—C10—N3	118.69 (16)
C2—C3—C4	118.98 (19)	C9—C10—C11	122.07 (16)
C4—C3—H3	120.5	C11—C10—N3	119.24 (16)
C3—C4—H4	120.7	C10—C11—H11	120.8
C3—C4—C5	118.57 (17)	C12—C11—C10	118.40 (15)
C5—C4—H4	120.7	C12—C11—H11	120.8
N1—C5—C4	123.20 (17)	C7—C12—H12	119.6
N1—C5—C6	115.24 (16)	C11—C12—C7	120.71 (16)
C4—C5—C6	121.55 (16)	C11—C12—H12	119.6
N2—C6—C5	121.55 (16)		
			
O1—N3—C10—C9	−5.2 (3)	C3—C4—C5—C6	−179.92 (17)
O1—N3—C10—C11	175.38 (18)	C4—C5—C6—N2	−1.8 (3)
O2—N3—C10—C9	174.23 (18)	C5—N1—C1—C2	0.1 (3)
O2—N3—C10—C11	−5.2 (3)	C6—N2—C7—C8	139.41 (19)
N1—C1—C2—C3	−0.1 (3)	C6—N2—C7—C12	−45.1 (3)
N1—C5—C6—N2	178.44 (17)	C7—N2—C6—C5	175.13 (15)
N2—C7—C8—C9	175.98 (17)	C7—C8—C9—C10	−1.4 (3)
N2—C7—C12—C11	−174.32 (16)	C8—C7—C12—C11	1.1 (3)
N3—C10—C11—C12	179.77 (15)	C8—C9—C10—N3	−178.34 (16)
C1—N1—C5—C4	0.0 (3)	C8—C9—C10—C11	1.1 (3)
C1—N1—C5—C6	179.76 (17)	C9—C10—C11—C12	0.3 (3)
C1—C2—C3—C4	−0.1 (3)	C10—C11—C12—C7	−1.5 (3)
C2—C3—C4—C5	0.2 (3)	C12—C7—C8—C9	0.3 (3)
C3—C4—C5—N1	−0.2 (3)		

### Hydrogen-bond geometry (Å, º)

**Table:**

*D*—H···*A*	*D*—H	H···*A*	*D*···*A*	*D*—H···*A*
C1—H1···O1^i^	0.93	2.89	3.510 (3)	125
C2—H2···O1^ii^	0.93	2.65	3.343 (3)	132
C6—H6···O2^iii^	0.93	2.65	3.527 (2)	158
C11—H11···N1^iv^	0.93	2.60	3.465 (2)	155

Symmetry codes: (i) *x*, *y*, *z*+1; (ii) *x*−1, *y*, *z*+1; (iii) *x*+1/2, −*y*+1/2, *z*+1/2; (iv) *x*−1/2, −*y*+1/2, *z*−1/2.
