# Supplementary figures and images for: Crystal structure of 4-nitro-N-[(pyridin-2-yl)methyl­idene]aniline
Source: Acta Crystallogr E Crystallogr Commun. 2015 Sep 17;71(Pt 10):o760. doi: 10.1107/S2056989015016928 (PMC4647417; doi:10.1107/S2056989015016928)

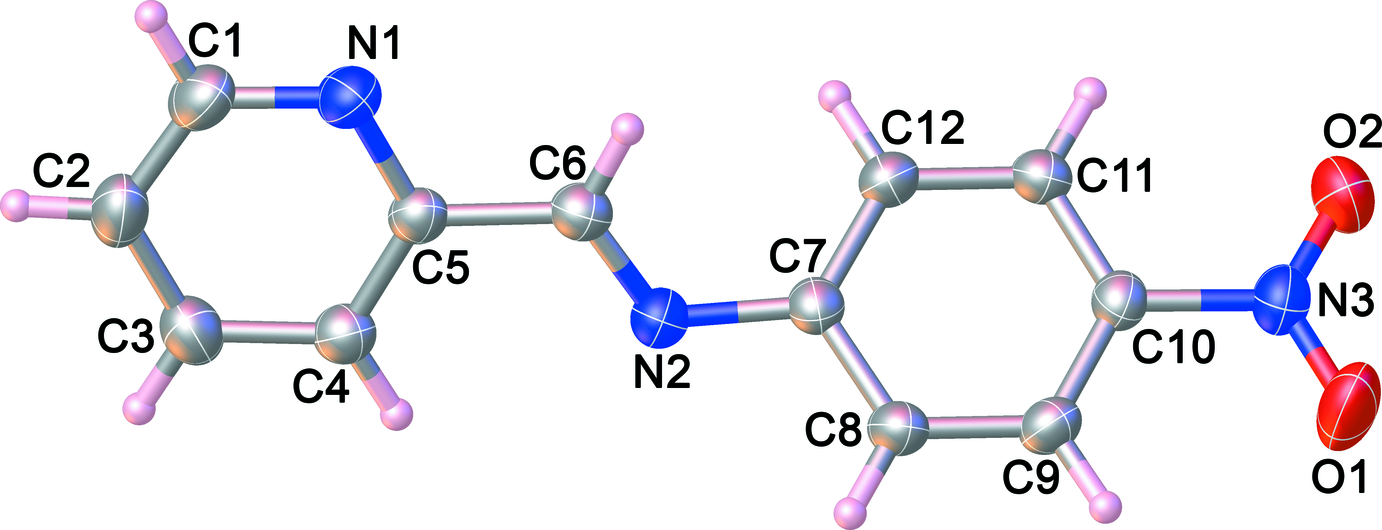

Supplement: Supplementary file 5 [file e-71-0o760-fig1.tif]

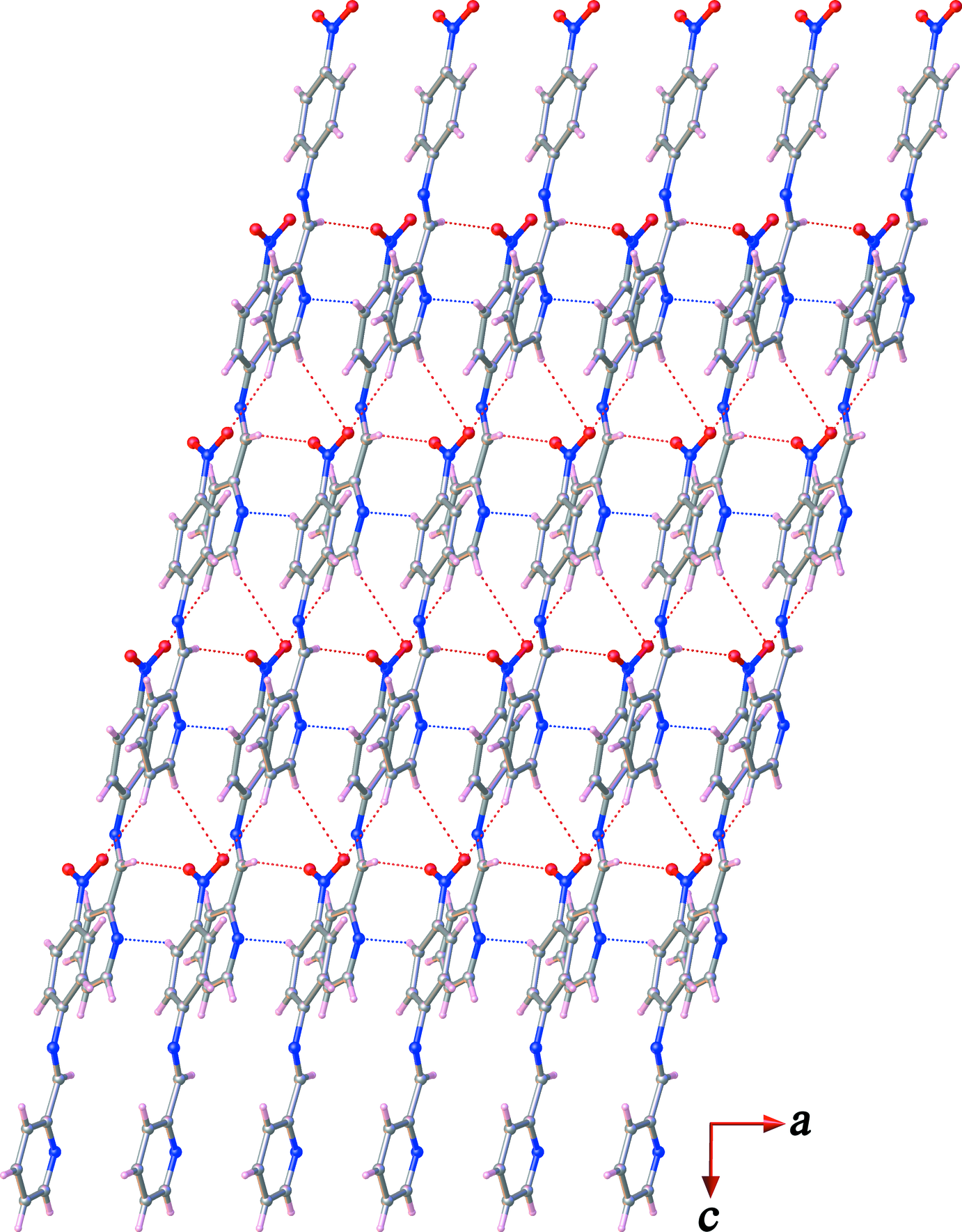

Supplement: Supplementary file 6 [file e-71-0o760-fig2.tif]
